# The trend of breeding value research in animal science: bibliometric analysis

**DOI:** 10.5194/aab-66-163-2023

**Published:** 2023-06-28

**Authors:** Fatma Yardibi, Chaomei Chen, Mehmet Ziya Fırat, Burak Karacaören, Esra Süzen

**Affiliations:** 1 Department of Animal Science, Akdeniz University, Antalya, Türkiye; 2 College of Computing and Informatics, Drexel University, Philadelphia, PA, USA; 3 Department of Electrical and Electronics Engineering, Akdeniz University, Antalya, Türkiye

## Abstract

This study aims to identify trends and hot topics in breeding value to support researchers in finding new directions for future research in that area. The data of this study consist of 7072 academic studies on breeding value in the Web of Science database. Network visualizations and in-depth bibliometric analysis were performed on cited references, authors, countries, institutions, journals, and keywords through CiteSpace. VanRaden (2008) is the most cited work and has an essential place in the field. The most prolific writer is Ignacy Misztal. While the most productive country in breeding value studies is the United States, the People's Republic of China is an influential country that has experienced a strong citation burst in the last 3 years. The National Institute for Agricultural Research and Wageningen University are important institutions that play a critical role in connecting other institutions. Also, these two institutions have the highest centrality values. “Genomic prediction” is the outstanding sub-study field in the active clusters appearing in the analysis results. We have summarized the literature on breeding value, including publication information, country, institution, author, and journal. We can say that hot topics today are “genome-wide association”, “feed efficiency”, and “genomic prediction”. While the studies conducted in the past years have focused on economic value and accuracy, the studies conducted in recent years have started to be studies that consider technological developments and changing world conditions such as global warming and carbon emission.

## 1 Introduction

Falconer and Mackay (1983) defined the breeding value of an individual as the average value of progeny, which is a simple but powerful concept in plant and animal breeding. The deviation of the progeny produced by a particular progenitor from the mean of the reference population is the breeding value (Ceballos et al., 2016). Falconer and Mackay (1983) defined an individual's breeding value not only in terms of the average performance of a reference population, but also in terms of the value of alleles it could pass from each ancestor to its progeny (Falconer and Mackay, 1983). It is generally accepted that breeding value is primarily influenced by additive genetic effects, which are the effects of genes passed down from parents to offspring and contribute to the individual's genetic makeup. However, other genetic effects such as dominance effects, epistatic effects, and gene–environment interactions can also contribute to the variation in breeding values observed within a population. An accurate estimation of a selection candidate's breeding value in a breeding program is crucial. It indicates the selected candidate's ability to breed superior progeny. It is clear that there will be a greater need to increase the efficiency of animal product production. The primary purpose of livestock farming is to produce goods and services for humanity's benefit using the feed materials of natural resources such as animal genotypes and the environmental conditions to which these genotypes are exposed. From this point of view, the size of the benefit or economic benefit from animals depends on that animal's genetic capacity. However, studying animals with the best genetic capacity for animal products may not always be possible. At this point, animal breeding plays a critical role in increasing the average genetic ability of the current population. The estimated breeding value can make it possible to rank the animals according to their predicted genetic potential, resulting in more accurate selection and faster genetic progression across generations.

A fundamental principle of animal breeding is to select animals as parents, which will improve the genetic level of the next generation. For quantitative traits for which genotypes can not be observed, the breeding value may measure the phenotypic value influenced by both the genotype and the environment (Toghiani, 2012). Animal breeding aims not to improve individual animals genetically but to improve animal populations. In animal breeding, the first condition of establishing a selection program is to know the genetic characteristics of the traits of interest (Toghiani, 2012).

Early prediction methods in breeding were based either on progeny testing or phenotypes measured in selection candidates. These methods were then expanded to selection indices which used various information sources, such as measurements of the same phenotype collected from relatives and combinations thereof and secondary traits measured in the same individual (Lopez-Cruz and De Los Campos, 2021). In the 1950s, C. R. Henderson further expanded the methodology, developing mixed models containing fixed and random effects. These methods have been used to identify the animals with the best genetic potential and, therefore, the highest value for breeding (breeding value), using information about phenotypes determined not only by the genetic potential but also by environmental influences. Henderson proposed the best linear unbiased predictor (BLUP) in 1984 to calculate estimated breeding values based on these phenotypic data.

Defining breeding goals is one of the critical challenges of a breeding organization. While breeding goals determine where to go, breeding programs describe how to get there (Simianer, 2021). Key in genetic improvement programs is the estimation of breeding values (EBVs). Breeding value, which can be defined as the genetic values of an individual specified by the progeny, may be based on individual characteristics or selection index. Suppose there is only a single record on one animal and no information on their relatives. In that case, the EBV is the heritability multiplied by the difference between the individual observation and the population mean (Pal and Chakravarty, 2019).

The principal objective of animal breeding is to generate genetic progress to breeding aims at changing populations in a desired, positive direction. Therefore, an important task of animal breeding research should be to enable genetic advancement.

There is a growing body of literature that recognizes the importance of molecular markers (such as single-nucleotide polymorphism – SNP) for genomic prediction of breeding values (Meuwissen et al., 2001). Genomic prediction of phenotypes and breeding values is an increasingly important area in animal science (Meuwissen et al., 2001; VanRaden, 2008a). For example, over the past two decades, major advances in genomic prediction have doubled the dairy cattle improvement due to reducing generation intervals (Hayes et al., 2009; de Koning, 2016).

The bibliographic analysis can explore the academic field of knowledge and understand which questions researchers are trying to answer and what methods they have developed for this purpose (Chen, 2014). This method involves using a defined set of metrics to assess published research output, impact, and trends. In other words, bibliometric analysis is used to qualitatively and quantitatively analyze the effects of journals, institutions, research groups, individual researchers, or countries (Kamdem et al., 2019).

Bibliometric studies summarize and analyze the current situation's changes, and research hotspots in breeding value studies are required to make an ideal research plan (Yardibi et al., 2021). This study's results will help researchers see the development of breeding value literature, understand its course, and conduct better-planned research. This study is aimed to provide benefits in terms of seeing the progress of studies in this field and better evaluating the question marks for the future.

## 2 Materials and methods

### 2.1 Methods

Bibliometric analysis was applied to investigate the origin, development, and evolution of the breeding value field, as well as the current status and possible trend (Wang et al., 2020; Yardibi et al., 2021). This bibliometric study used CiteSpace, one of the most popular software tools to analyze co-citation networks (CiteSpace V 5.8.R3 update on 4 January 2022 and CiteSpace V 6.1.R2 advanced update on 10 May 2022; software available at https://citespace.podia.com/, last access: 7 December 2022). Visualization maps used for bibliometric analysis in CiteSpace consist of nodes and links. While nodes represent analytical items such as author, journal, reference, and keyword, the node size shows the total co-occurrence frequency of an item, the node's thickness, and the ring's color indicates the co-occurrence time periods of this item (Chen et al., 2012). Colored lines indicate connections between different nodes, describing collaboration and co-occurrence or co-citations. The thickness of a line between other nodes indicates the frequency of citations together. In contrast, the color of the lines indicates the first year of co-citation relationships between these nodes (Chen et al., 2012). We used three structural measures to evaluate the network's structural quality: modularity Q index, mean silhouette score, and betweenness centrality value. The modularity Q index means the divisibility rating of a network into smaller components. If the modularity Q index is high, network clusters have fewer inter-cluster overlaps (Chen, 2006); if the modularity Q value is Q > 0.3, the cluster structure is defined as important. The mean silhouette score measures the quality of clusters and cluster homogeneity (Chen, 2006). If the silhouette values are > 0.5, the cluster structure is homogeneous. If the silhouette values are > 0.7, the clusters obtained are considered reliable (Chen, 2006). The betweenness centrality is measured by a node's ability to connect with other nodes, which is another crucial index (Chen et al., 2010). To give an example, a node with a high value of betweenness centrality (value between 0 and 1) illustrates that the node represents a critical point linking two or more groups that shows a transition pattern and indicates the primary topics in a network (Chen et al., 2010). The centrality value exceeding 0.1 has more impact, and the higher the frequency, the greater the influence (Su et al., 2019). Besides the structural metrics, temporal metrics were also examined for the analysis of nodes. The first metric in this group is citation burst, which is an important point in the research field. A citation burst is defined in the literature as a keyword, author, institution, or document that changes with sudden frequency over time (Li et al., 2021). Another metric is sigma, obtained by considering betweenness centrality and citation burst simultaneously (Chen et al., 2010). The timeline view finally provides an overview of the evolution of clusters in the field over time and shows whether these developments continue over the years (Lin et al., 2020).

### 2.2 Data collection

In this study, the data for bibliometric analysis are taken from the well-known academic database website Web of Science (WoS) database, a publisher-independent platform that provides comprehensive citation information for various academic disciplines (Lin et al., 2020; Hou et al., 2021). Web of Science “core collection” was preferred rather than all databases to obtain full records (title, author, publication information, abstract, and reference) of influential papers and eliminate the subordinate papers. Web of Science core collection (update: 4 January 2022) data, consisting of 7072 publications, had been downloaded. Search criteria were selected using the following search terms: topic – animal breeding or breeding value or selection index or selection intensity or breeding efficiency or breeding program; categories – agriculture, dairy, and animal science; document type – article or review article or proceeding paper; and time span – 2000–2021. According to the determined criteria, 7072 papers' data were reached and recorded appropriately. The recorded data were converted into Excel data and were checked. It was then converted to a data file format suitable for CiteSpace, saved, and analyzed. The analysis adopted the g -index selection criteria with a scale factor k set at 25. The g index, based on the h index, is the “largest number that equals the average number of citations of the most highly cited g publications” (Egghe, 2006). The selection criteria and the values of the scaling factors were chosen after several trials that aimed at optimizing the network structure metrics.

**Figure 1:**
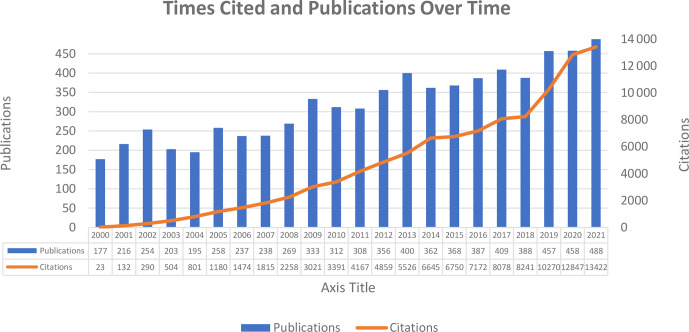
Publication and citation distribution.

## 3 Results

### 3.1 Analysis of publication output

A total of 7072 publications on breeding value have been published, and the detail of the annual publication is shown in Fig. 1. As shown in Fig. 1, the sum of times cited per year was just 23 in 2000. Since then, the number of citations per year has increased faster than in previous years. Further, the increase in the number of publications can be seen in Fig. 1.

It can be said that the number of publications in an increasing trend is an indicator that the field is still developing. The increasing trend in the number of citations is faster than the increasing trend in the number of publications.

The h index of the field was 102, which is the index of scientific research impact, and the average citation per item value was 14.88, calculated automatically on WoS. Of 7072 documents, approximately 94 % consists of articles, 2 % are reviews, and 4 % are proceedings papers.

### 3.2 Analysis of leading countries and institutions

**Figure 2:**
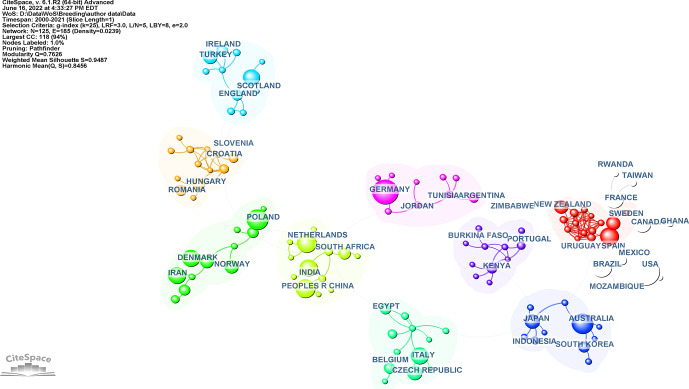
Visualization of country collaboration network is represented as countries publishing breeding value studies from 2000–2021. Nodes on the map represent countries. Lines between nodes represent cooperative relationships: the larger the area, the more active the country.

The country collaboration network's map consists of 125 nodes and 185 links. Figure 2 shows the countries and cooperation networks of these authors. Each circle represents the country's outputs, and each link describes their collaboration, as shown in Fig. 2.

The top 10 countries in terms of the number of publications and centrality are listed in Table 1. The United States was the most productive country in the breeding value studies ( n : 885, 12.51 %). The countries with the highest centrality were Germany (0.20), the United States (0.17), France (0.12), Italy (0.12), and Australia (0.11), which were the main centers of country collaboration worldwide.

Table 1 shows the number of publications, centrality values, and mean (years) of the countries and institutions.

The network's density is calculated as 0.0239. Furthermore, the burstness is a valuable metric for determining which country is particularly active; as shown in Table 2, the People's Republic of China has had a strong burst over the last 3 years. According to Table 2, the People's Republic of China, South Korea, and Brazil have been the most active countries in recent years. As a result of cluster analysis for countries, the modularity Q value is relatively high (0.7626). The structures are reasonable enough; this means that the clusters are not partially nested and separated. Cluster analysis results for countries were not given.

Table 2 shows the top 10 countries and top 10 institutions with the citation bursts and their years of popularity. The red line indicates the active citation burst duration between 2000 and 2021, whereas the blue line corresponds to the inactive duration.

**Figure 3:**
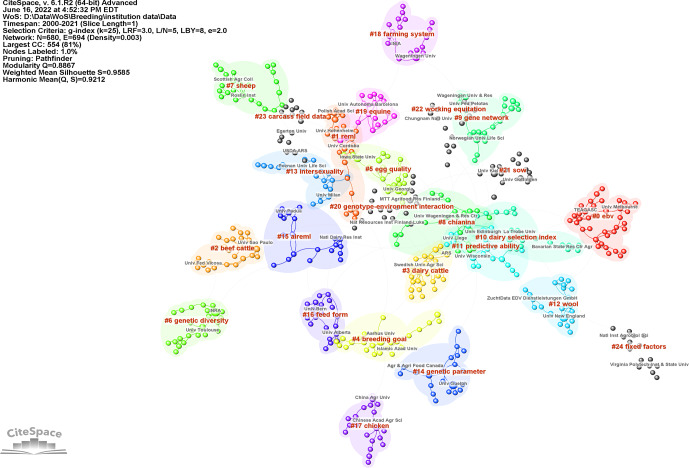
Maps of institutions cited in the literature from 2000 to 2021 on breeding value: cluster map of institutions. The name of the cluster represents the topics of the institutions. CiteSpace configuration: LRF = 3, LBY = 8, L/N = 5, e = 2.0, and g index ( k = 25). Network: 680 institutions and 694 collaboration links.

The institution's collaboration network's map consists of 680 nodes and 694 links. Figure 3 shows the institution and cooperation cluster networks. Cluster refers to a group of related publications that share similar characteristics for institutions. Each circle represents the institution's outputs and each link describes its collaboration. We can understand from the density of connections that the most active institutions cooperate with each other. As a result of the cluster analysis for institutions, the modularity Q value was 0.8867, and the mean silhouette value was 0.9585. As a result of these two important metrics that define the general structural features of the network, it can be said that this network is divided into homogeneous and cluster reliability (see in Fig. 3). The cluster of estimated breeding value represented by no. 0 is the largest. It means that the cluster has the most members (institutions).

As shown in Table 1, the most active institution was found at the University of Wageningen, followed by National Institute for Agricultural Research (INRA) and the University of Guelph. Moreover, the University of Wageningen and INRA are key nodes with the highest betweenness centrality value (respectively 0.19 and 0.15) because the centrality is greater than 0.1, which means they play an important role in connecting other institutions. The University of Toulouse and Northwest A&F University are today's active institutions with a strong citation burst, as shown in Table 2.

**Figure 4:**
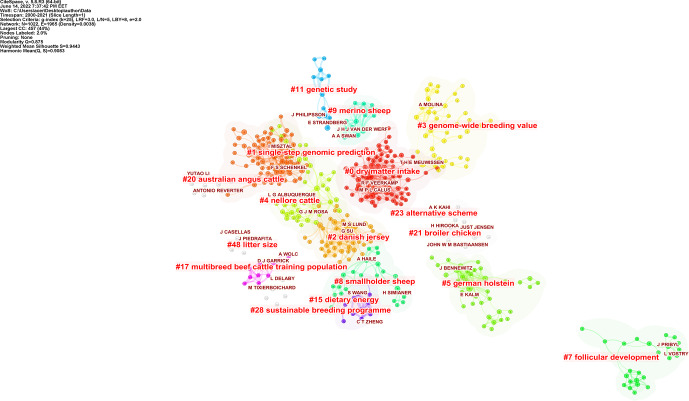
Visualization of the author cluster structures. The name of the cluster represents the topics of the authors. CiteSpace configuration: LRF = 3, LBY = 8, L/N = 5, e = 2.0, and g index ( k = 25). Network: 1022 authors and 1965 collaboration links.

### 3.3 Analysis of the journals

A total of 7072 academic studies on breeding values were published in 146 different scientific journals. The journal with the highest number of publications was the *Journal of Animal Breeding and Genetics* with 1124 (15.89 %) academic studies, followed by *Archives of Animal Breeding* with 920 (13.01 %), *Journal of Dairy Science* with 713 (10.08 %), *Journal of Animal Science* with 420 (5.94 %), and *Genetics Selection Evolution* with 391 (5.53 %). In other words, approximately 50 % of the academic studies in this field have been published in these five journals. The journals' co-citation map is a complex network consisting of 1231 nodes and 13070 links. According to the analyses of this network, the *Journal of Animal Science* had the maximum co-citation counts (3880); next was the *Journal of Dairy Science* (3667). These journals are “core journals” with high publication and co-citation volume.

**Table 1:** The top 10 countries in terms of publications numbers and top 10 institutions publishing on the association breeding value between 2000–2021.

Counts ${}^{*}$	Centrality	Year	Countries
885	0.17	2000	USA
718	0.20	2000	Germany
467	0.11	2000	Australia
465	0.04	2000	Poland
418	0.06	2000	Brazil
402	0.03	2001	People's Republic of China
394	0.09	2000	Netherlands
350	0.11	2000	Spain
344	0.12	2000	France
320	0.12	2000	Italy
Counts ${}^{*}$	Centrality	Year	Institution
311	0.19	2000	University of Wageningen
233	0.15	2000	National Institute for Agricultural Research
232	0.14	2005	University of Aarhus
198	0.12	2000	University of New England
193	0.14	2000	University of Guelph
150	0.07	2000	USA Department of Agricultural Research Service
145	0.06	2000	Swedish University Agriculture Science
136	0.09	2000	University of Georgia
130	0.05	2001	University of Iowa State
128	0.07	2000	University of Edinburgh

${}^{*}$ Counts: number of academic studies published by countries and institutions.

**Table 2:**
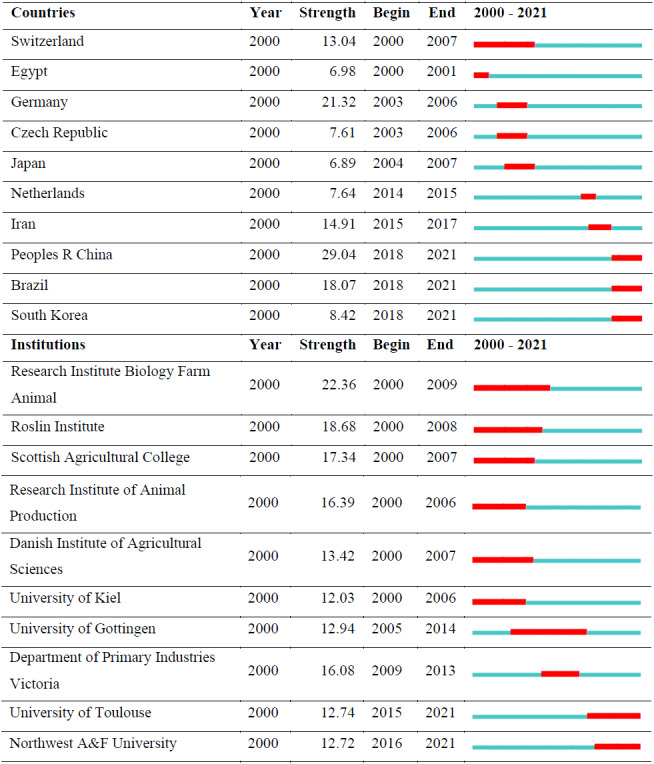
Country and institution citation bursts.

### 3.4 Analysis of the authors

There were 14 115 different authors in 7072 papers. The 10 most active and prolific authors and their quantitative metrics are listed in Table 3.

Ignacy Misztal was the most productive researcher, with 79 papers in this field. As shown in Table 3, the following research by Legarra et al. (2009), of which Misztal is a co-author, “A relationship matrix including full pedigree and genomic information”, and Hayes et al. (2009), “Invited review: Genomic selection in dairy cattle: Progress and challenges”, plays an important role in breeding value studies. Author T. H. E. Meuwissen has the highest centrality value (0.08) compared to other authors. Therefore, it is a key point in connecting with other authors. The author network consists of 1022 nodes and 1965 links. As a result of the cluster analysis of the network, the modularity Q value was 0.875, and the silhouette value was 0.9443. Figure 4 shows the cluster analysis map of the network. It means that with metric values close to 1, the network is divided into reasonably homogeneous reliable clusters.

**Table 3:** Top 10 active and prolific authors and their quantities metrics with their co-authorship.

Author	Counts ${}^{*}$	Centrality	Most cited study	Citation	Co-authorrelationship
Misztal, I.	79	0.07	A relationship matrix including full pedigree and genomic information (Legarra et al., 2009)	461	Legarra, A.Aguilar, I.Misztal, I.
Calus, M. P. L.	66	0.03	Reliability of direct genomic values for animals with different relationships within and to the reference population (Pszczola et al., 2012)	178	Pszczola, M.Strabel, T.Mulder, H. A.Calus, M. P. L.
Veerkamp, R. F.	61	0.04	Dairy cattle breeding objectives combining yield, survival and calving interval for pasture-based systems in Ireland under different milk quota scenarios (Veerkamp et al., 2002)	94	Veerkamp, R. F.Dillon, P.Kelly, E.Cromie, A. R.Groen, A. F.
Lund, M. S.	51	0.06	Genomic prediction for Nordic Red Cattle using one-step and selection index blending (Su et al., 2012)	91	Su, G.Madsen, P.Nielsen, U. S.Maentysaari, E. A.Aamand, G. P.Christensen, O. F.Lund, M. S.
Meuwissen, T. H. E.	51	0.08	Using the genomic relationship matrix to predict the accuracy of genomic selection (Goddard et al., 2011)	189	Goddard, M. E.Hayes, B. J.Meuwissen, T. H. E.
Berry, D. P.	49	0.02	Genetics of feed efficiency in dairy and beef cattle (Berry and Crowley, 2013)	197	Berry, D. P.Crowley, J. J.
Schenkel, F. S.	49	0.04	A genome scan to detect quantitative trait loci for economically important traits in Holstein cattle using two methods and a dense single-nucleotide polymorphism map (Daetwyler et al., 2008)	105	Daetwyler, H. D.Schenkel, F. S.Sargolzaei, M.Robinson, J. A. B.
Legarra, A.	47	0.04	A relationship matrix including full pedigree and genomic information (Legarra et al., 2009)	461	Legarra, A.Aguilar, I.Misztal, I.
Pryce, J. E.	46	0.03	Genetics and genomics of reproductive performance in dairy and beef cattle (Berry et al., 2014)	171	Berry, D. P.Wall, E.Pryce, J. E.
Hayes, B. J.	45	0.03	Invited review: Genomic selection in dairy cattle: Progress and challenges (Hayes et al., 2009)	1028	Hayes, B. J.Bowman, P. J.Chamberlain, A. J.Goddard, M. E.

${}^{*}$ Counts: the number of publications by the author on the breeding value between the selected years.

**Table 4:** Author analysis and cited author analysis of the top 10 large-size clusters summary.

Author analysis cluster ID	Cluster size	Silhouette	Mean (year)
No. 0 Dry matter intake	83	0.891	2011
No. 1 Single-step genomic prediction	77	0.959	2014
No. 2 Danish Jersey	49	0.910	2008
No. 3 Genome-wide breeding value	44	0.975	2007
No. 4 Nellore cattle	42	0.910	2008
No. 5 German holstein	38	0.930	2003
No. 7 Follicular development	25	0.979	2002
No. 8 Smallholder sheep	24	0.985	2009
No. 9 Merino sheep	16	0.996	2011
No. 11 Genetic study	13	0.998	2004
Cited author analysis cluster ID	Cluster size	Silhouette	Mean (year)
No. 0 Dairy cow	259	0.721	2008
No. 1 Genomic prediction	229	0.804	2014
No. 2 Residual feed intake	155	0.739	2009
No. 3 Genetic diversity	151	0.797	2008
No. 4 QTL mapping	117	0.848	2005
No. 5 Pedigree analysis	116	0.813	2006
No. 6 Racing performance	25	0.964	2007
No. 7 Growing pig	19	0.939	2014
No. 8 Friesian herd	15	0.982	2007
No. 9 Water-holding capacity	10	0.992	2008

Author analysis examined authors of 7072 academic studies on breeding value, and cited author analysis also examined authors cited in those studies. The cluster summary is given in Table 4. The software does not display the names of clusters with low silhouette values in the cluster summary table, clusters visualization, or timeline (Chen et al., 2010). The silhouette value of all clusters was greater than 0.7; therefore, the clusters were well clustered in a homogeneous structure. Clusters no. 0 (dry matter intake) and no. 1 (single-step genomic prediction) are still actively studied sub-field topics.

Table 4 shows the cluster size, silhouette values, and mean (years) of the clusters automatically selected.

**Table 5:**
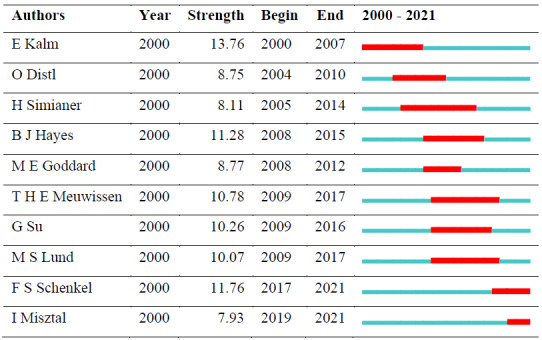
Top 10 authors with the citation bursts.

The citation burst, which is considered an important point in the research field, is given in Table 5 for the authors. A burst term is defined as an author who changes in literature with a sudden frequency during a given period.

E. Kalm made the strongest citation burst between 2000 and 2007. The most active author, I. Misztal, is in the top 10 in the citation explosion with his works between 2019 and 2021, and his works belong to cluster no. 1 (single-step genomic prediction). B. J. Hayes, M. P. L. Calus, R. F. Veerkamp, Bijma P, J. E. Pryce, and M. E. Goddard, active and influential authors in the field, belong to the no. 0 (dry matter intake) cluster.

Table 5 shows the top 10 authors with citation bursts and their years of popularity. The red line indicates the active citation burst duration between 2000 and 2021, whereas the blue line corresponds to the inactive duration.

According to the cited author analysis result, T. H. E. Meuwissen (citation count: 1061) ranked first, followed by P. M. VanRaden (citation count: 992), and I. Misztal (citation count: 727). The field of study of these three authors belongs to cluster no. 1 genomic predictions determined from the cited author analysis (see Table 4). The highly co-cited authors are known to significantly influence the development of a particular field. Therefore, it can be said that these authors made an important contribution to the development of the breeding value field.

**Table 6:** Top 20 keywords with centrality value.

Rank	Counts ${}^{*}$	Centrality	Year	Keywords
1	1022	0.02	2000	selection
2	962	0.02	2000	trait
3	822	0.02	2000	genetic parameter
4	794	0.03	2000	dairy cattle
5	622	0.02	2000	cattle
6	618	0.01	2000	parameter
7	600	0.02	2000	breeding value
8	572	0.02	2000	growth
9	503	0.03	2000	prediction
10	482	0.02	2000	performance
11	459	0.02	2000	population
12	426	0.02	2000	beef cattle
13	415	0.01	2000	model
14	357	0.02	2000	genetic evaluation
15	331	0.01	2000	cow
16	328	0.03	2000	information
17	318	0.01	2007	genomic selection
18	313	0.01	2000	milk yield
19	303	0.02	2001	accuracy
20	275	0.02	2000	milk production

${}^{*}$ Counts: the number of times the keyword was seen.

### 3.5 Analysis of the keyword

The co-occurrence of keywords can effectively reflect research hotspots and boundary issues and provide research support (Kiliçaslan et al., 2021). The keyword network's map consists of 756 nodes and 9549 links. It means that, on average, each determined keyword was connected with 12.65 other keywords. The 20 most frequently used keywords are shown in Table 6.

**Table 7:**
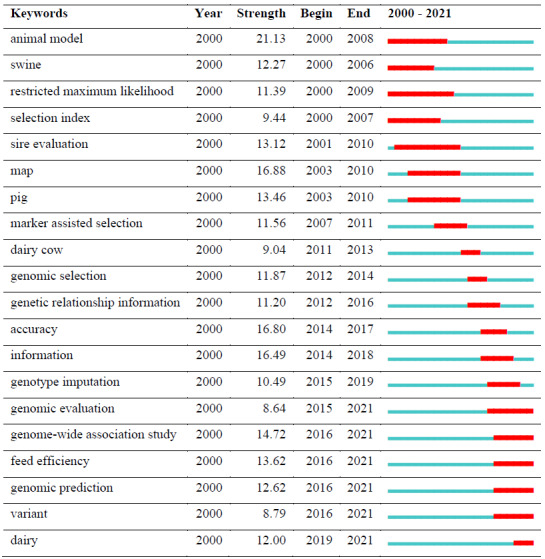
Top 20 keywords with the strongest citation bursts.

The most popular keyword was “selection” ( n : 1022) regarding the frequency. The modularity Q value was 0.3259, the mean silhouette was 0.6696, and seven clusters were defined. It cannot be said that the structures of the clusters are homogeneous enough and relatively reliable, since the silhouette value is less than 0.7, and the modularity Q value is greater than 0.3 but very close. “Burst citation” was determined as an indicator of research frontier topics in a certain time period. There have been a total of 246 citation bursts, and the top 20 keywords with the citation bursts are shown in Table 7.

Table 7 shows the top 20 keywords with the highest citation bursts and their years of popularity. The red line indicates the active citation burst duration between 2010 and 2021, whereas the blue line corresponds to the inactive duration.

In addition, the keywords “genome-wide association”, “feed efficiency”, “genomic prediction”, “variant”, and “dairy” had citation bursts in recent years. From 2000 to the present, the strongest citation bursts in keywords giving a general idea of research trends by showing authors' focus and achievements were “animal model”, “map”, and “accuracy”. The current findings support the relevance of animal models with associated mapping information to improve prediction accuracy.

**Table 8:** 10 cited references with centrality value

Counts ${}^{*}$	Centrality	Year	Cited References
283	0.07	2008	VanRaden, P. M., 2008, *Journal of Dairy Science* (VanRaden, 2008)
238	0.07	2009	VanRaden, P. M., 2009, *Journal of Dairy Science* (VanRaden et al., 2009)
211	0.05	2009	Hayes, B. J., 2009, *Journal of Dairy Science* (Hayes et al., 2009)
187	0.02	2010	Aguilar, I., 2010, *Journal of Dairy Science* (Aguilar et al., 2010)
170	0.10	2009	Goddard, M., 2009, *Genetica* (Goddard, 2009)
161	0.00	2009	Gilmour, A., 2009, ASReml User Guide (Gilmour et al., 2009)
135	0.02	2010	Christensen, O. F., 2010, *Genetics Selection Evolution* (Christensen and Lund, 2010)
120	0.05	2007	Habier, D., 2007, *Genetics* (Habier et al., 2007)
118	0.01	2014	Sargolzaei, M., 2014, *BMC Genomics* (Sargolzaei et al., 2014)
114	0.01	2011	Habier, D., 2011, *BMC Bioinformatics* (Habier et al., 2011)

Counts: The number of times the cited references was seen.

### 3.6 Analysis of the reference and co-citation

The co-citation principle is used as a research tool to calculate the degree of relationship between documents (Kiliçaslan et al., 2021). Analysis of CiteSpace is conducted not only by considering the documents downloaded from databases but also by analyzing the references they have cited in their text. Therefore, the document co-citation analysis generates a network that consists of both cited documents and citing (the ones downloaded from scientific platforms) (Carollo et al., 2021). The co-citation network of a total of 116 624 cited references related to 7072 academic studies consisted of 1546 nodes and 7531 links, and each determined document was connected with 4.87 other references. The first 10 most cited academic studies are given in Table 8.

P. M. VanRaden's article “Efficient Methods to Compute Genomic Predictions” in 2008 (VanRaden, 2008) was referenced in 283 of the selected breeding value studies, and the article was cited 2610 times in total. At the same time, this article with the strongest citation burst has the feature of being an interesting article in a short time. Another P. M. VanRaden academic study in 2009, “Invited Review: Reliability of genomic predictions for North American Holstein bulls” (VanRaden et al., 2009), was referenced 238 times in breeding value studies. B. J. Hayes's 2009 review “Invited review: Genomic selection in dairy cattle: Progress and challenges” (Hayes et al., 2009) was referenced 211 times and cited 1028 times. The reference with the greatest centrality values is the “Positional candidate cloning of a QTL in dairy cattle: identification of a missense mutation in the bovine DGAT1 gene with major effect on milk yield and composition” article by Grisart et al. (2002) (centrality: 0.26 and Σ : 83.52). This article is significant, as it is the first successful positional cloning effort of a quantitative trait loci (QTL) in an outbred species, including humans. We can say that it is a key point between other nodes.

**Figure 5:**
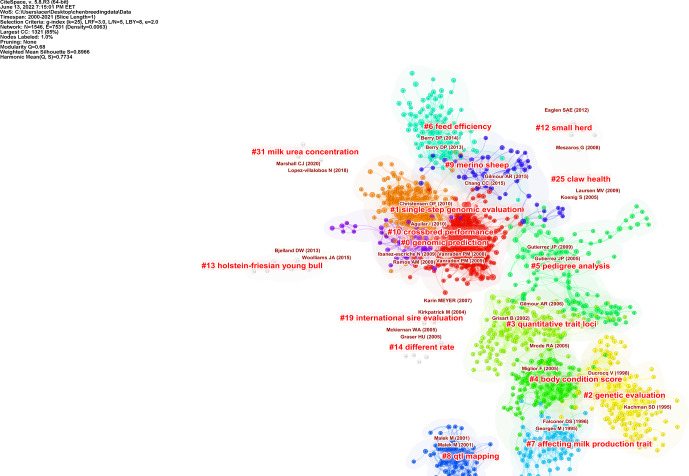
Document co-citation analysis of a cluster's structure. The significant clusters view reference maps cited in the literature from 2000 to 2021. The smaller the number, the more nodes the cluster contains. The name of the cluster represents the topics of the references. CiteSpace configuration: LRF = 3, LBY = 8, L/N = 5, e = 2.0, and g index ( k = 25). Network: 1546 references and 7531 co-citation links.

**Table 9:** The top 11 clusters summarized according to references.

Cluster ID	Cluster size	Silhouette	Mean (year)
No. 0 Genomic prediction	264	0.797	2010
No. 1 Single-step genomic evaluation	186	0.858	2014
No. 2 Genetic evaluation	139	0.945	1998
No. 3 Quantitative trait loci	131	0.890	2003
No. 4 Body condition score	115	0.926	2000
No. 5 Pedigree analysis	115	0.907	2004
No. 6 Feed efficiency	100	0.953	2014
No. 7 Affecting milk production trait	77	0.959	1998
No. 8 QTL mapping	61	0.990	1999
No. 9 Merino sheep	49	0.929	2012
No. 10 Crossbred performance	48	0.929	2013

As a result of cluster analysis, the modularity Q value is 0.680, and the silhouette value is 0.896. The results are reliable, as the quality indicator's silhouette value and modularity Q meet the criteria. The cluster map of the co-citation reference analysis is shown in Fig. 5, and the 10 largest clusters identified in the co-citation reference analysis and their metrics are shown in Table 9.

The major clusters identified in the co-citation reference were homogeneous relatively (see Fig. 5 and Table 9).

Table 9 shows the cluster size, silhouette values, and mean (years) of the clusters automatically selected.

**Figure 6:**
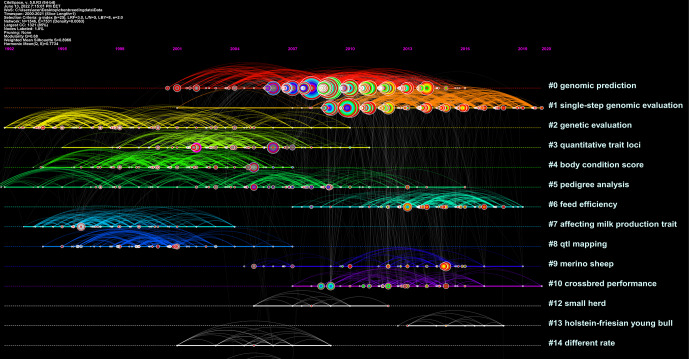
Document co-citation analysis of a cluster's timeline. The name of the cluster means the topics of cited authors. Lines between the nodes represent co-citation relationships

The cluster no. 0 genomic prediction is the largest cluster, consisting of 264 nodes. It had a silhouette score of 0.797, which is relatively low in homogeneity. The references composing cluster were, on average, published in 2010. The second-largest cluster no. 1 (single-step genomic evaluation) was a group of 186 nodes with a silhouette score of 0.858 and a publication year that, on average, was 2014. The third-largest cluster, no. 2 (genetic evaluation), was a group of 139 nodes with a high silhouette score of 0.945 and was, on average, published in 1998. As seen in Fig. 6, the timeline view would finally provide an overview of the evolution of clusters in the breeding value field over time and show whether these improvements have continued over the years. Single-step genomic evaluation and feed efficiency clusters are active sub-fields (see Fig. 6).

**Table 10:** Top 20 references with the strongest citation bursts.

Reference	Strength of	Year	Beginning of	End of	Burst	Sigma	Centrality
	burstness		burstness	burstness	duration		
Efficient methods to compute genomic predictions (VanRaden, 2008)	55.05	2008	2011	2016	5	48.41	0.07
A new approach for efficient genotype imputation using information from relatives (Sargolzaei et al., 2014)	38.88	2014	2016	2021	5	1.44	0.01
ASReml user guide (Gilmour et al., 2009)	38.12	2009	2013	2017	4	1.00	0.00
Prediction of total genetic value using genome-wide dense marker maps (Meuwissen et al., 2001)	30.74	2001	2007	2009	2	1.42	0.01
The impact of genetic relationship information on genome-assisted breeding values (Habier et al., 2007)	29.65	2007	2009	2015	6	3.70	0.05
Invited review: Genomic selection in dairy cattle: Progress and challenges (Hayes et al., 2009)	29.50	2009	2010	2015	5	4.03	0.05
Introduction to quantitative genetics (Falconer, 1996)	28.01	1996	2000	2004	4	10.75	0.09
Strategy for applying genome-wide selection in dairy cattle (Schaeffer, 2006)	27.72	2006	2007	2013	6	3.02	0.04
Invited review: Reliability of genomic predictions for North American Holstein bulls (VanRaden et al., 2009)	27.36	2009	2010	2013	3	5.62	0.07
Manual for BLUPF90 family of programs (Misztal et al., 2014)	26.77	2014	2019	2021	2	1.05	0.00
Single Step, a general approach for genomic selection (Legarra et al., 2014)	25.46	2014	2017	2021	4	1.51	0.02
A unified approach to utilize phenotypic, full pedigree, and genomic information for genetic evaluation of Holstein final score (Aguilar et al., 2010)	23.28	2010	2016	2018	2	1.57	0.02
ASReml user guide release 4.1 structural specification (Gilmour et al., 2015)	22.66	2015	2017	2021	4	1.10	0.00
Selection indices in Holstein cattle of various countries (Miglior et al., 2005)	22.09	2005	2006	2012	6	1.53	0.02
Changes in genetic selection differentials and generation intervals in US Holstein dairy cattle as a result of genomic selection (García-Ruiz et al., 2016)	21.86	2016	2017	2021	4	1.37	0.01
Second-generation PLINK: Rising to the challenge of larger and richer datasets (Chang et al., 2015)	20.89	2015	2018	2021	3	1.12	0.01
Genome-wide association mapping including phenotypes from relatives without genotypes (Wang et al., 2012)	20.70	2012	2017	2021	4	1.36	0.01
Genomic prediction when some animals are not genotyped (Christensen and Lund, 2010)	20.36	2010	2014	2018	4	1.45	0.02
ASReml user guide release 2.0 (Gilmour et al., 2006)	20.23	2006	2008	2013	5	1.02	0.00
Genomic selection: Prediction of accuracy and maximisation of long term response (Goddard, 2009)	19.92	2009	2010	2017	7	6.60	0.10

In the co-citation reference network, 476 nodes indicated a citation burst in their history (see Table 10 for the strongest 20 citation burst). In particular, in the first 10 nodes with the highest magnitude of citation bursts, eight belonged to cluster no. 0 (genomic prediction). It was seen that Gilmour et al. have three strong citations burst for different ASReml user guide versions among the top 20 citations burst. ASReml, a statistical package that fits linear mixed models using residual maximum likelihood (REML), has been given references by many authors. It can be said that linear mixed model structures have been frequently preferred in breeding value studies since the 2000s. The second document with the strongest citation burst, “A new approach for efficient genotype imputation using information from relatives,” was authored by Sargolzaei et al. (2014).

At the same time, this document has the most up-to-date citation burst with a duration of 5 years (from 2016 to 2021). In the sigma metric, the document with the highest value was the one published by VanRaden (2008), with a value of 48.1. A high sigma node not only has a strategically important structural feature but also has unique temporal implications (Chen, 2016).

## 4 Discussion

This study aimed to analyze the current status and trends between 2000 and 2021 publications on breeding value using CiteSpace. This bibliometric analysis has shown that relevant research about breeding value was grown from year to year. The increase in the number of citations that is faster than the increase in the number of publications indicates that the citations given to the publications made in this field are shown not only in the area but also in other fields. This can also be explained by breeding value studies being a multidisciplinary field that includes genetics, statistics, biotechnology, and other related fields. In other words, breeding value studies are also cited in secondary fields. The studies of VanRaden, Meuwissen, and Misztal, the most cited authors in studies on breeding value, are of great importance for those working in this field. These studies played a key role in the development of the field. P. M. VanRaden's 2008 study “Efficient Methods to Compute Genomic Predictions” is a significant work in the breeding value field, cited 4155 times. He is a prominent author who has developed efficient methods for processing genomic data to improve the accuracy of estimated breeding value and simultaneously predict thousands of marker effects. Another study by P. M. VanRaden in 2009, “Invited review: Reliability of genomic predictions for North American Holstein bulls,” has been cited 1394 times. B. J. Hayes's 2009 study “Invited review: Genomic selection in dairy cattle: Progress and challenges” is another important study that has been cited 1878 times. The highest betweenness centrality belongs to Mike Goddard's academic study “Genomic selection: prediction of accuracy and maximization of long term response” in 2009. Additionally, this document has a long period of citation burst (2010–2017). He studied the accuracy estimation of genomic selection and maximizing long-term response and noted that unless new markers were continually added to the estimation of breeder value, genomic selection would result in a faster reduction in selection response than phenotypic selection (Goddard, 2009). The most prolific author I. Misztal's article “A relationship matrix including full pedigree and genomic information” has great importance in the field. Although the United States was the most productive country in the breeding value studies ( n : 885, 12.51 %), the People's Republic of China has had a strong burst over the last 3 years. The most active institutions were found at Wageningen University and INRA. At the same time, the University of Wageningen and the INRA have the highest betweenness centrality that plays an important role in connecting other institutions. “Breeding scheme” and “sustainable breeding program” clusters are the current sub-areas that institutions work with intensively.

The linear BLUP approach, BayesA, and BayesB, among the statistical models proposed to estimate the genomic breeding value, were extensively studied from 2001 to 2010 (Meuwissen et al., 2001; VanRaden, 2008b; Meuwissen and Goddard, 2004; Muir, 2007; Villumsen et al., 2009). However, Gianola and Fernando (1986) provided an elegant and critical review of Bayesian methods applied in animal breeding before the Markov chain Monte Carlo (MCMC) era, with their work making an important contribution to Bayesian methodology applied to theoretical animal breeding and quantitative genetics. Based on the number of references to ASReml by many authors, we can also say that regression models and linear mixed models are generally used as statistical methods in breeding value studies. Strong cooperation has been identified between many developed countries and well-known institutions. Although it is difficult to publish relevant articles in high-impact journals, breeding value studies have been kept up to date and have high co-citation counts. When compared with the journals where the most influential articles were generally published, it was found that the most cited articles on breeding value were published in *Journal of Animal Science*, *Journal of Dairy Science*, *Genetic Selection Evolution*, *Journal of Animal Breeding and Genetics*, and *Livestock Production Science*.

A more in-depth analysis of the 10 prolific authors and the 10 strongest citation bursts showed that the most common topics mainly focused on genomic prediction, single-step genomic prediction, and dry matter intake. Based on the assumption that hotspot analysis is the basis for assessing the evolution of keywords in breeding value, we can say that hot topics today are “feed efficiency” and “genomic prediction”. The “genomic prediction” is the essential sub-study field in the active clusters that appears in the analysis results. Since its initial development in the early 2010s, the single-step genomic best linear unbiased predictor has become the most popular methodology for genetic evaluations, including for genotyped and non-genotyped individuals. It has accurately estimated breeding values in animals and plants. A new breeding value has been studied in the last decade, combining the amount of feed saved through improved metabolic efficiency with the expected maintenance requirements (Pryce et al., 2015). Therefore, breeding value involves a genomic component for residual feed intake (RFI) with maintenance requirements calculated from a genomic or pedigree estimated breeding value (EBV) for body weight (BW) estimated using adaptation traits (Pryce et al., 2015). Today's whole-genome sequencing studies have created a strong need for faster and more scalable applications of basic functions such as logistic regression, genomic distance evaluation, and linkage disequilibrium estimation. C. C. Chang et al.'s 2015 article “Second-generation PLINK: rising to the challenge of larger and richer datasets” addresses this need (Chang et al., 2015). It is also an indication of this that this document has a strong and up to date citation burst.

As a matter of course, when it comes to animal breeding, the names of the pioneers of animal breeding such as Henderson who developed the best linear unbiased predictions and mixed models and Lund should not be forgotten. Because the time period of this study does not include the years before 2000, that is, the active years of such pioneers' names, it is not directly included in this study. Our primary aim in this study is to focus on how animal breeding will take a direction in the future, recognizing that it has come from a certain point rather than a complete historical development. Consequently, this study aimed to assess the importance of breeding value in animal science using various bibliometric analyses. “Genomic prediction” provided the largest set of important clusters over various research topics associated with breeding value estimation. It is only since the work of Meuwissen et al. (2001) that the study of genomic selection or prediction has gained momentum. The main theoretical assumptions and associated statistical models to obtain the benefits of genomic prediction were first demonstrated by Meuwissen et al. (2001) by using simulated datasets. Genomic relationship matrices to obtain genomic breeding values were first defined by (VanRaden, 2009). However, the impact of different genomic relationship matrices (referring genetical architecture of the phenotypes) on the accuracy of genomic prediction of breeding values has remained unclear (Misztal et al., 2020). From this standpoint, the popularity of the “genomic prediction” cluster could be understandable, and further research in this field should be carried out to obtain breeding values with higher accuracy under various experimental settings.

In the mid-20th century, technological innovations in computation and methodological advances in genetic theory and statistics paved the way for powerful multi-trait analysis. As more sophisticated analytical techniques for traits were developed and incorporated into selection programs, production began to increase rapidly, and the wheels of genetic progress began to turn (Miglior et al., 2017). The evolution of breeding value studies into genetic breeding value over the past 20 years has been with the adoption of the idea of using DNA markers to improve the genetic gain rate. In this study's time period considered, it is seen that the developments in the first 10 years are static; however, the developments in the last 10 years are more dynamic. The way has been opened for better genetic predictions with the identification of single genes or the quantitative trait loci (QTL). Many of the economically important traits in livestock are complex, continuously distributed phenotypes influenced by multiple polygenes located at QTL distributed throughout the genome (Grisart et al., 2002). Remarkable advances in production efficiency have been made following the application of sophisticated selection strategies based on quantitative genetic theory.

All aspects of animal health and welfare have an additional non-monetary value that cannot be measured economically. This extra value has two interrelated aspects: the suffering of affected animals and the social impact. A merely economic approach will never capture the full value of such features (Simianer, 2021). While the studies conducted in the past years have focused on economic value and accuracy, the studies conducted in recent years have started to be studies that take into account technological developments and changing world conditions such as global warming. We believe that, in the future, international commitments and coordinated action to limit global warming directly related to greenhouse gas emissions, nutrient efficiency, and climate adaptation will play a more critical role in breeding goals than a purely economic approach. Ultimately, it is possible to evaluate and optimize breeding programs with different objective functions (Simianer, 2021). Defining cultivation goals is one of the critical entrepreneurial challenges of a cultivation organization. It always has to consider the expected future production conditions, demand structures, and socio-economic context. Breeding goals will likely have to respond to society's expectations of animal welfare and growing skepticism about the over-industrialization of animal production (Hernandez et al., 2022). The livestock sector's new goals should consider reducing greenhouse gas emissions, environmental protection, and climate change. Climate change, environmental impact mitigation, and animal adaptation are current emerging issues in the livestock industry, and these will bring new breeding objectives and new research areas (Cassandro, 2020).

## 5 Conclusion

Although technological developments since the beginning of the last century have facilitated the calculation of breeding value, developments in genetic theory have increased the complexity of calculations. With the development of technology, even more data will be available for use in selection (such as data loggers, on-farm sensors, and precision measuring techniques). The problem will be determining which traits to choose from within this dataset, which contains many traits, rather than measuring phenotypes. It is clear that future studies will seek an answer to the question of which traits should be included in the breeding value. It is already know that the focus of selection had shifted from being purely production oriented toward a more balanced breeding goal. This change has partly been due to increasing health and fertility problems and partly to social pressure and welfare concerns. In addition to selection indices such as longevity, fertility, and health for sustainable breeding goals, studies that integrate features such as reducing greenhouse gas emissions and protecting the environment into selection indices will increase rapidly in the future.

As a result, it is possible to say that future studies will focus on innovative issues that may have limited environmental impacts while reducing costs and accelerating livestock productivity.

Nevertheless, combining bibliometrics and visual knowledge maps provides researchers with a reliable way to review the literature, helping them comprehensively and systematically understand the development and evolution of hotspots in a given field. This study aims to comprehensively present the studies on breeding values and examine the studies' development stages and potential trends. As a result, this study can be a pioneer in this field by revealing important research countries and institutions, influential journals, leading authors, cooperation between countries and institutions, general trends, and hot topics and can provide an excellent guide for further studies.

## Data Availability

All data for this article were downloaded from the public Web of Science database. The datasets used and analyzed during the current study are available from the corresponding author upon reasonable request.
